# A novel composite of CdS nanorods growing on a polyaniline-Cd^2+^ particles surface with excellent formaldehyde gas sensing properties at low temperature

**DOI:** 10.1039/c8ra05082a

**Published:** 2018-08-31

**Authors:** Ling Zhang, Xifeng Li, Zonggang Mu, Jing Miao, Kun Wang, Rui Zhang, Shunquan Chen

**Affiliations:** School of Water Conservancy and Environment, University of Jinan Jinan 250022 China chm_zhangl@ujn.edu.cn +86 53189736287; School of Chemistry and Chemical Engineering, University of Jinan Jinan 250022 China chm_lixf@ujn.edu.cn +86 53182765475; Shenzhen Institute of Advanced Technologies, Chinese Academy of Sciences (CAS) Shenzhen 518055 China; Guangdong Key Laboratory of Membrane Materials and Membrane Separation, Guangzhou Institute of Advanced Technology, CAS Guangzhou 511458 China jing.miao@giat.ac.cn +86 20 22912757; Department of Safety, Health and Environmental Engineering, National Kaohsiung First University of Science & Technology Kaohsiung City 824 Taiwan

## Abstract

A novel composite, CdS nanorods growing on a polyaniline-Cd^2+^ particles surface (CdS/PANI) with a hexagonal wurtzite structure phase, was prepared using a hydrothermal synthesis method. Methods of XRD, SEM, and FTIR were used to analyze the structure and morphology of the compounds. SEM shows that CdS/PANI consists of sea urchin-like nanorods of about 200–500 nm in length and about 50 nm in diameter. Furthermore, the FTIR spectra show that some characteristic peaks of CdS/PANI are much weaker than those of PANI and the corresponding peaks shift to a higher wavenumber. In addition, the IR stretching frequency of the Cd–S bond for CdS/PANI moved from 630 cm^−1^ to 674 cm^−1^. In the gas sensing experiments, the CdS/PANI-based sensor showed an excellent response to low concentration formaldehyde gas in a wide temperature range of 80–140 °C. The highest response of CdS/PANI could reach about 4.8 to 5 ppm formaldehyde gas at 120 °C. The response and recovery times of the sensor based on CdS/PANI were about 25 s and 30 s to 10 ppm formaldehyde gas, respectively.

## Abstract 2

A novel composite, CdS nanorods growing on a polyaniline-Cd^2+^ particles surface (CdS/PANI) with a hexagonal wurtzite structure phase, was prepared using a hydrothermal synthesis method.

## Graphical abstract

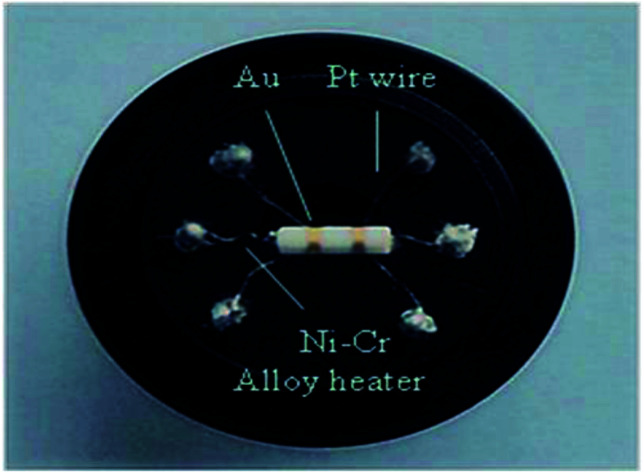

## 1. Introduction

Formaldehyde (HCHO) commonly exists in indoor decorative materials and is one of the most serious pollutants and carcinogenic volatile organic compounds. Exposure to formaldehyde can cause central nervous system damage, blood, immune system and developmental disorders, blindness and respiratory disease. Hence, the efficient detection of HCHO is of great importance and much needed for human health.

Semiconducting sensors can monitor this kind of gas quickly and are inexpensive as well. For example, CdS is an important II–VI group semiconductor compound with a narrow band gap (∼2.42 eV), which has attracted much research interest due to its excellent transport properties, good thermal and chemical stability and controllable morphology.^1,2^ It has also been reported that CdS is a good sensing material with ultrafast response and recovery speed for alcohols, NO_2_, alcoholic chain gases *etc.*^3–8^ Inorganic composites of CdS materials also show very good gas-sensing properties. For example, CdS/ZnO heterostructures have shown significant performance for visible light induced photoelectric gas sensing of formaldehyde.^9^ PbS/CdS nanowires with a nano-heterojunction show good sensing properties to liquefied petroleum gas (LPG) at room temperature due to the advantages of adsorbing and de-adsorbing LPG molecules easily by penetrating from top to bottom.^10^

Besides inorganic semiconducting sensors, organic conductive polymers such as polyaniline (PANI) based sensors also have been well established due to the facile synthesis, simple acid/base doping/de-doping chemistry and high gas sensing response.^11^ Polyaniline-based electrochemical gas sensors have shown fast responses to various organic vapors also have been widely used to detect combustible, toxic gases such as LPG and toluene and triethylamine vapor.^12–15^ Sensors based on PANI/inorganic nanocomposites were also widely investigated and many significant results were obtained. It was reported that p-polyaniline/n-TiO_2_ with heterojunction had good response to liquefied petroleum gas (LPG) at room temperature.^16^ Sensors based on flexible SiO_2_/PANI nanofibers were reported showing very good response and excellent selectivity for ammonia gas at room temperature.^17^ ZnO-PANI nanocomposite was reported showing better gas sensing efficiency as compared to the ones from single phase PANI film.^18^ PANI/ZnO hybrid film with p–n junction, which provided efficient gap for gas diffusion and abundant adsorption sites, have been developed for ammonia (NH_3_) detection at room temperature^19^ and can detect VOCs at low temperature.^20^ Polyaniline coated graphene hybridized SnO_2_ nanocomposite was reported showing gas response to ammonia at room temperature.^21^ Sensors based on Al–SnO_2_/PANI composite nanofibers (fabricated *via* electrospinning technique) shown to be a hydrogen sensor with high-efficiency.^22^ More of that, compared with PANI, composites of PANI/TiO_2_, PANI/SnO_2_ and PANI/In_2_O_3_ thin films all had good selectivity and long-term stability to NH_3_ gas.^23^ Recently, B. T. Raut *etc.* reported a sensor based on polyaniline-CdS nanocomposites fabricated by a simple spin coating technique and the sensor was observed to have an considerable response to H_2_S than the singles.^24^ D. S. Dhawale *etc.* also found that the sensors based on n-CdS/p-polyaniline with heterojunction exhibits high response towards LPG room temperature.^25^ They thought that the nanocomposites of PANI and CdS may form a p–n junction and the increasing response of the nanocomposites may be due to the creation of positively charged depletion layer on the surface of CdS which could be formed by the inter-particle electron migration from CdS to PANI at the junction.

However, the above conducting polymer/inorganic nanocomposites were often prepared by methods of physical mixing. We think that in this way the two phases may not been dispersed evenly and which may be hinder the sensor from showing its best performance. In this work, we adopted a simple chemical synthesis process, *i.e.*, hydrothermal synthesis, to make CdS crystals grown on the PANI-Cd^2+^ particles surface. In this way, a novel CdS nanorods growing on the surface of polyaniline-Cd^2+^ particles surface (labeled as CdS/PANI) with sea urchin-like morphology was prepared. We found that the CdS/PANI sample-based sensors have excellent formaldehyde gas sensing properties at low temperature. CdS/PANI-based sensor showed very excellent response to low concentrations of formaldehyde gas in a wide temperature range of 80–140 °C, the highest response of CdS/PANI could reach about 4.8 to 5 ppm formaldehyde gas at 120 °C. The response and recovery time of the sensor based on CdS/PANI was about 25 s and 30 s to 10 ppm formaldehyde gas, respectively.

## 2. Experimental

### 2.1 Reagents and materials

All chemicals were of AR grade, and were used as received. Aniline was distilled before use.

### 2.2 Synthesis

#### (1) The synthesis of CdS nanorods

CdS nanorods were synthesized using a simple hydrothermal process. First, quantitative amount of Cd(NO_3_)_2_·4H_2_O and thiourea at mass ratios of 90 : 70, were stirred well in reactors lined with PTFE. Then ethylenediamine was added to fill 60% volume of the reactor. Second, the reactors were placed in an incubators at 160 °C for 48 hours to form two types of yellow precipitates, *i.e.*, CdS nanorods. In the last step, the precipitates were washed with ethanol and deionized water several times to remove organic solvent and inorganic ions before drying at 60 °C for 10 hours. The resultant CdS composite was ready for test.

#### (2) The synthesis of CdS/PANI composites nanorods

First, adding a quantity of polyaniline (CAS: 5612-44-2) to a certain concentration of cadmium nitrate solution, stirring thoroughly, filtering and drying to obtain PANI-Cd^2+^. Then quantitative amount of Cd(NO_3_)_2_·4H_2_O, thiourea and PANI-Cd^2+^ at mass ratios of 90 : 70 : 2, were stirred well in reactors lined with PTFE. The following steps are the same as the preparation of CdS nanorods, and then resultant CdS/PANI composite was prepared for test.

### 2.3 Characterization of materials

X-ray powder diffraction (XRD) patterns of the samples were recorded on a Rigaku D/Max-2200X powder X-ray diffraction meter using Cu K radiation at 40 kV and 40 mA in the range of 20° to 80° (2*θ*) with a scan speed of 8° min^−1^. The surface morphologies of PANI, CdS and CdS/PANI were studied by means of scanning electron microscopy (SEM). Fourier transform-infrared spectra (FTIR) of the samples were recorded in KBr pellets with Bruker FTIR.

### 2.4 Measurement of sensors

**Fig. 1:**
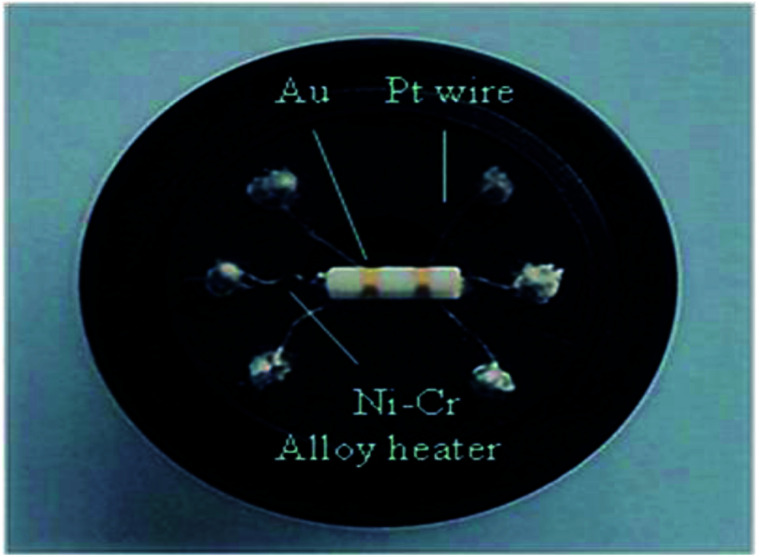
Photograph of a blank gas sensor.

The powder of the obtained sample was mixed with a suitable amount of deionized water and ground into paste. Then the paste was coated onto small ceramic tubes with a thickness of about 10 μm to form a thick-film gas sensor. The ceramic tubes were mounted with Au electrodes and Pt lead wires at both ends. Ni–Cr alloy wires as coil heating were inserted in the tubes to control the operating temperature. Fig. 1 shows the photograph of the fabricated sensor in the experiments. The gas sensing performance of the sensors were measured by CGS-8 intelligent gas sensing analysis system (Beijing Elite Tech Co., Ltd., China). The vapor samples are all analytically pure.

The prepared sensors were first dried for 24 hours in air at room temperature and then pre-heated at different operating temperatures for about 60 minutes, then aged the pipes at 90 °C for 12 hours before gas sensing tests. Fresh air (25–30% relative humidity at room temperature) was used as carrier gas. When the sensors' resistance in air was stable, a suitable amount of test gas was injected into the test chamber (20 L in volume). Mean values were obtained from the results of 5 different sensors and each test repeated three times.

The gas response in the experiments was defined as *R*_air_/*R*_gas_ (for n-type semiconductor) or *R*_gas_/*R*_air_ (for p-type semiconductor), where *R*_air_ is the sensor's resistance in air, while *R*_gas_ is that in a sample gas. The response and recovery times were defined as the times taken by the sensors to achieve 80% of the total resistance change after the ambience was changed.

## 3. Results and discussion

### 3.1 X-ray diffraction analysis

**Fig. 2:**
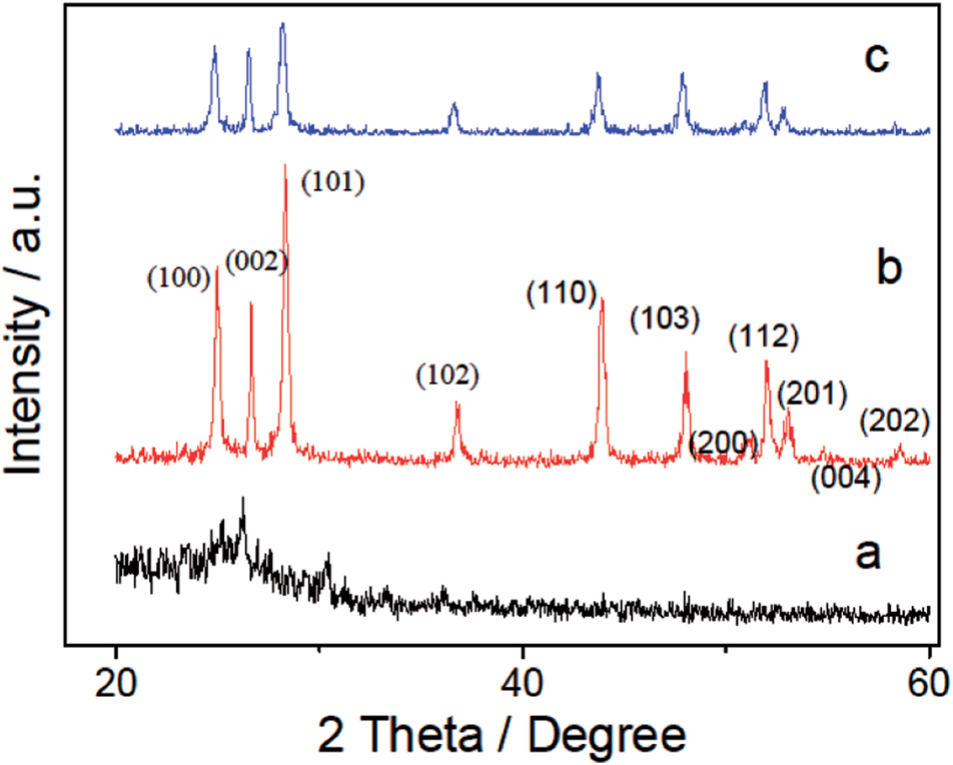
The XRD patterns of PANI (a), CdS (b) and CdS/PANI (c) samples.

The XRD patterns of PANI (a), CdS (b) and CdS/PANI (c) samples are shown in Fig. 2. The weak peaks at 2*θ* = 20.78° and 25.04° in PANI are attributed to (020) and (200) crystal planes of PANI (Fig. 2a). The main diffraction peaks of the CdS (b) sample indicate that CdS is crystallized in hexagonal wurtzite structure (JCPDS no. 77–2306, CdS *a* = 4.141A°, *c* = 6.720A°). As can be seen from Fig. 2b and c, diffraction peaks of the single CdS at 2*θ* angles of 25.0°, 26.4°, 28.3°, 36.7°, 43.9°, 48.0°, 51.2°, 52.1°, 53.1°, 54.8°, 58.6° can be indexed as the (100), (002), (101), (102), (110), (103), (200), (112), (201), (004) and (202) planes of the hexagonal wurtzite structure of CdS, respectively. It can be found that the diffraction peak of CdS (Fig. 2b) are sharper than that of the CdS/PANI (Fig. 2c), which suggested that the crystallinity and diameter sizes of CdS growing on the surface of polyaniline-Cd^2+^ particles surface were changed due to the influence of PANI. The slightly worse crystallinity and smaller diameter size of CdS in the composite may bring more defects and then the composite may obtain better gas sensing performance than pure CdS.

### 3.2 Surface morphology study

**Fig. 3:**
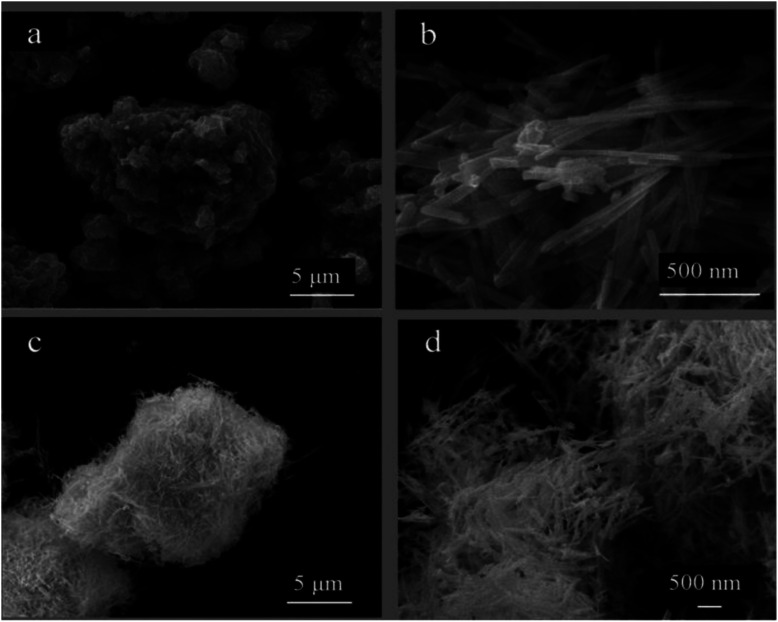
The surface micrographs of PANI (a), CdS (b) and CdS/PANI (c and d).

Surface morphologies of PANI, CdS and CdS/PANI have been studied by means of scanning electron microscopy (SEM). Fig. 3 shows the surface micrographs of PANI (a), CdS (b) and CdS/PANI (c and d). PANI (Fig. 3a) composites showed a agglomerate particle morphology with lamellar structure and the particle size is approximately 2–15 mm. Pure CdS (Fig. 3b) showed slender cylindrical shape or rod-like morphology with about 200–500 nm in length and 50 nm in diameter. As for CdS/PANI, it looked like a sea urchin, which showed reunions of fine needle or fuzzy morphology without obvious PANI layered granule. Obviously, CdS nanorods growing on base of polyaniline-Cd^2+^ particles surface have larger specific surface area than that of pure CdS and this morphology change may lead to the improvement of gas-sensing performance.

### 3.3 Fourier transform-infrared spectra

**Fig. 4:**
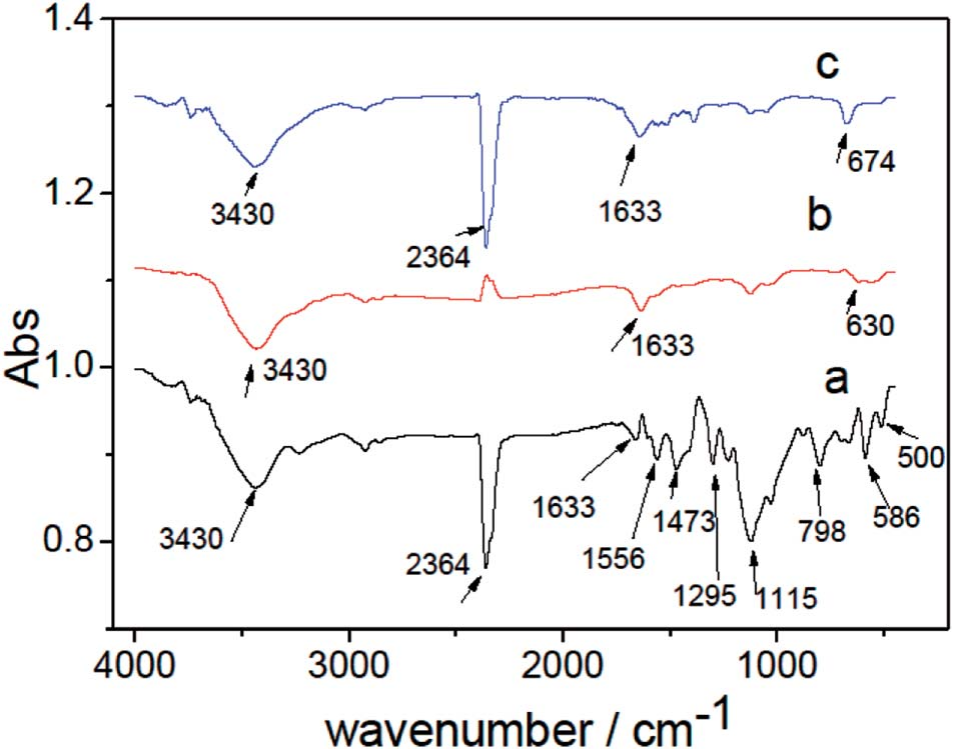
The FTIR spectra of PANI (a), CdS (b) and PANI/CdS (c) samples.

Fig. 4 shows the FTIR spectra of PANI, CdS and CdS/PANI samples. Broad peaks appeared at 3430 cm^−1^ and 1633 cm^−1^ are respectively attributed to O–H stretching and O–H bending modes which should be the result of the strong interaction of water with PANI, CdS and CdS/PANI.^26^ The strong peak at 2364 cm^−1^ is ascribed to the adsorption of CO_2_ of PANI and CdS/PANI samples, which shows has strong capacity for gas at room temperature.

Specially for polyaniline (Fig. 4a), the peak of the polyaniline shows characteristic peaks of emeraldine salt. In the spectrum, the peaks at 1473 cm^−1^ and 1556 cm^−1^ are assigned to the C=C stretching vibration mode of the quinonoid and benzenoid rings, while the bands at 1295 cm^−1^ is correspond to C–N stretching. The peaks of 1115 cm^−1^ and 798 cm^−1^ are C–H the flexural vibration absorption of out-plane and in-plane of the benzene ring;^27^ the peaks at 500 cm^−1^ is assigned to aromatic ring bending vibration.^28^

**Fig. 5:**
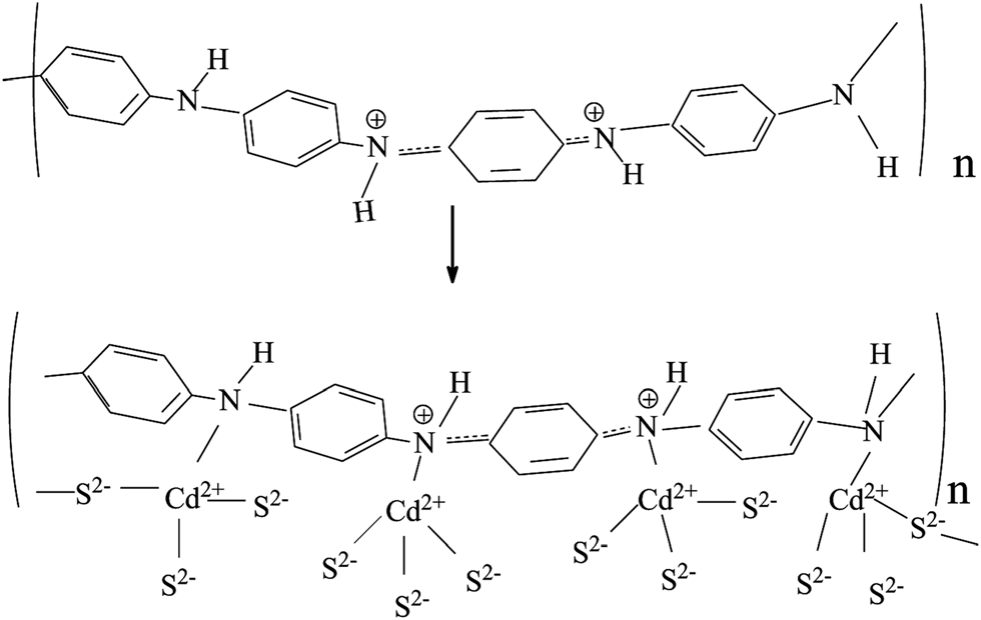
Schematic diagram of coordination bond between Cd^2+^ and amino of PANI.

For single CdS, Fig. 4b shows the characteristic peaks at 630 cm^−1^ which is ascribed to the stretching frequency of Cd–S bond.^29,30^ As for CdS/PANI, it is interesting to note that the FTIR spectrum (Fig. 4c) of CdS/PANI showed some important differences when compared with that of PANI and single CdS. On one hand, the intensity ratio of certain peaks of benzenoid ring, such as aromatic ring bending vibration peaks, C–N stretching and C–H flexural vibration absorption band, are much weaker than PANI and the corresponding peaks are shifted to higher wave number and which indicates that amino-group may be connected with Cd^2+^*via* coordination bond. On the other hand, the FTIR spectrum of CdS/PANI showed that its' stretching frequency of Cd–S bond shifted from 630 cm^−1^ to 674 cm^−1^ compared to that of CdS as shown in Fig. 5, which may be due to the coordination bond between Cd^2+^ and amino-group of PANI. According to the splitting mode of positive tetrahedron field, when S^2−^ ions are combined with Cd^2+^, the five d-orbitals of Cd^2+^ ion will divide and form a positive tetrahedron. However, at the boundary of Polyaniline and CdS, Cd^2+^ ions are combined with amino besides of S^2−^. Considering the differences of atomic radius and electro-negativity between N and S elements, the Cd^2+^ tetrahedron at the interface should be slightly out of shape. So the Cd–S bond energy may be slightly changed due to the deformed tetrahedron structure. This suggest that the CdS/PANI may be a new material through the coordination between Cd^2+^ and amino from PANI. Thus we consider that CdS/PANI in our work is probably different from the ones obtained by simple physical mixing ways.

### 3.4 Electric properties and formaldehyde-sensing characteristics

**Fig. 6:**
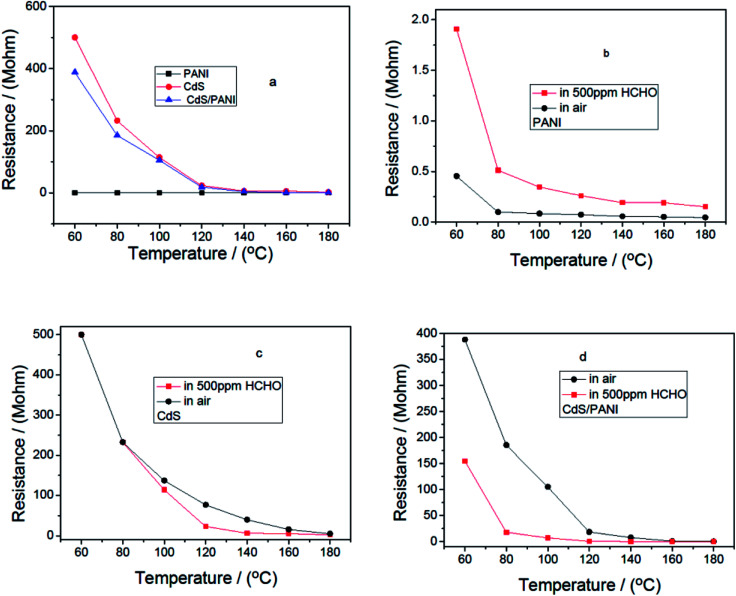
Resistance–temperature relationships of the sensors from different samples in air and in 500 ppm HCHO gas.

Fig. 6 shows the resistance behavior against temperature for sensors based on PANI, CdS and CdS/PANI samples in air and in 500 ppm HCHO gas. In the whole temperature range, the resistance values of all the sensors reduced with temperature increasing, which is the intrinsic characteristic of a semiconductor. From Fig. 6a, we also found that the resistance of PANI is the lowest; CdS is the highest at same temperature. This shows that the combination of PANI and CdS helps to improve the conductive ability of CdS.

Fig. 6b–d displayed that the resistance value of PANI sensor in 500 ppm HCHO were greater than that in the air. Contrarily, the resistance value of CdS and CdS/PANI sensors were less than that in air. It suggested that PANI is p-type semiconductor,^11^ while samples of pure CdS and CdS/PANI were n-type semiconductor.

From Fig. 6c, it can be seen that CdS has a certain response to formaldehyde gas, which may be caused by the density of states for CdS matching with the HOMO–LUMO levels of the target formaldehyde molecules.^31,32^ From Fig. 6d, it is also shown that CdS/PANI has much higher response to formaldehyde gas in low temperature, which suggested that the combination of CdS and PANI might have led to much better the matching between the density of states for CdS and the HOMO–LUMO levels of the target formaldehyde molecules than that of pure CdS.

**Fig. 7:**
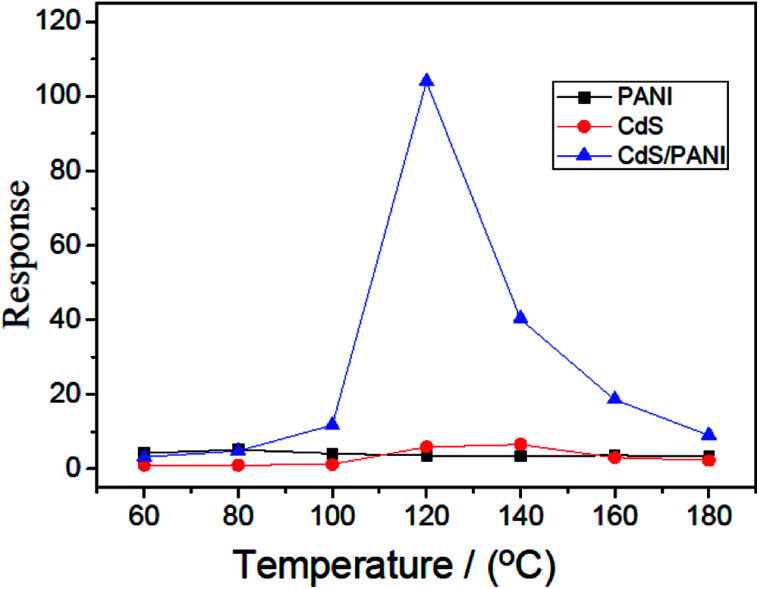
Response to 500 ppm HCHO gas of PANI, CdS and CdS/PANI sample-based sensors at different temperatures.

Fig. 7 shows the dynamic curves of the response of PANI, CdS and CdS/PANI sample-based sensors to 500 ppm HCHO gas at different operating temperatures. According to the dynamic curve, it is seen that CdS/PANI sample-based sensor exhibits very high response to HCHO gas in wide temperature range of 60–180 °C and the optimum operating temperature is about 120 °C with maximum response of 103.9. Besides of that, the sensor based of PANI also showed good response to 500 ppm HCHO gas. It is very interesting that an almost linear behavior of in response to 500 ppm HCHO gas for PANI in whole testing temperature from 60 °C to 180 °C. The response reached from 3.42 to 5.23. The sensor based on CdS nanorods shows fine response to 500 ppm HCHO gas at optimum working temperature 140 °C with maximum response 6.59.

**Fig. 8:**
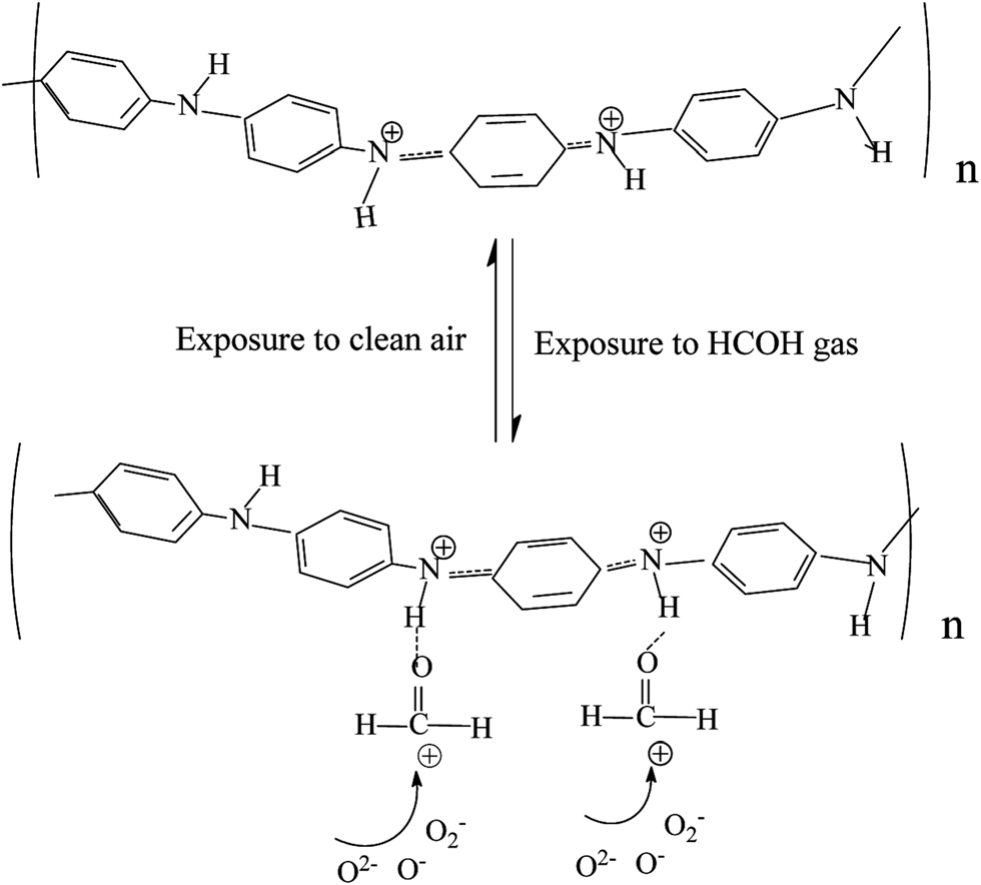
Schematic representation of gas sensing mechanism for PANI and CdS/PANI.

The mechanism of this gas sensing process can be explained as follows: PANI is a p-type semiconductor which means that its conductivity comes from hole carrier. Formaldehyde is an electron donor gas. When formaldehyde adsorbed on the surface of the sensing film, it delivered electron to the large amount of amino and gets ionized on the surface as showed in Fig. 8. This resulted in decrease in the density of the hole carrier in PANI backbone, such as solitons, polarons and bipolarons,^33^ which reduced conductive charge carrier concentration, thus the response behavior to the formaldehyde gas was showed. In addition, this adsorption mechanism can help to understand the almost level linear behavior of in response to HCHO gas for PANI in whole testing temperature from 60 °C to 180 °C as shown in Fig. 7.

For CdS nanorods, their gas sensing process involves charge exchange and gas molecule adsorption and desorption on the sensing element. In normal atmosphere, oxygen molecule will first adsorb on the surface of the sensing film and then trap the electrons of CdS to produce different negative oxygen species, including (O_2_^−^, O^−^ and O^2−^) by capturing free electrons from CdS. When the CdS sensor is exposed to formaldehyde gas, the reducing gas and the chemisorbed oxygen on the surface of CdS can give rise to the following reaction.(HCHO)_ads_ + 2(O_ads_)^−^ = CO_2_ + H_2_O(g) + 2e^−^

During the process, electrons will be released back to the sensing materials following the reactions placed below. As a result, the depletion region thickness of the sensing materials will decrease which will lead to increase in conductivity, *i.e.* the sensor shows response behavior to the test gas.

For CdS/PANI, its gas sensing response, however, was significantly higher than that of single PANI and CdS. Possible causes can be explained as follows: first, the reduction of crystallization may lead to more lattice defects, that means, more conducive to gas adsorption capacity. Secondly, the smaller size of the material could increase the specific surface area, which is conducive to the adsorption of gas. Third, the coordination between Cd^2+^ and amino of PANI causes the small deformation of the CdS lattice, which leads to the change of the Cd–S bond energy. The change in Cd–S bond energy may cause a change in the adsorption properties of CdS/PANI to gases.

Moreover, for CdS/PANI, in normal atmosphere, the benzene ring in polyaniline is rich in electrons, which is very conducive to oxygen molecules adsorption on the one hand; on the other hand, CdS also have a strong ability to absorb oxygen. So a large number of oxygen species (O_2_^−^, O^−^ or O^2−^) has been produced on the surface of the material by capturing free electrons from CdS/PANI which result in more formaldehyde molecules to be oxidized reached on the surface of CdS/PANI by molecular diffusion. This process will increase the electron density of crystallite CdS/PANI and then more oxygen species (O_2_^−^, O^−^ or O^2−^) are produced and result in more formaldehyde molecules to be oxidized reached on the surface of CdS/PANI by molecular diffusion. Thus the sensor based on CdS/PANI nanorods shows different response characteristics from pure CdS and PANI.

The dependence of the response on the concentration of HCHO gas at their respective optimum working temperature is shown in Fig. 8. When the concentration of HCHO gas increases, the response of sensors PANI, CdS and CdS/PANI also increases. We can see from Fig. 8 that there are low response to low concentrations of HCHO gas for the sensors of PANI and CdS nanorods, but for the CdS/PANI nanorods based sensor, there is a very high response to each low concentrations of HCHO gas.

**Fig. 9:**
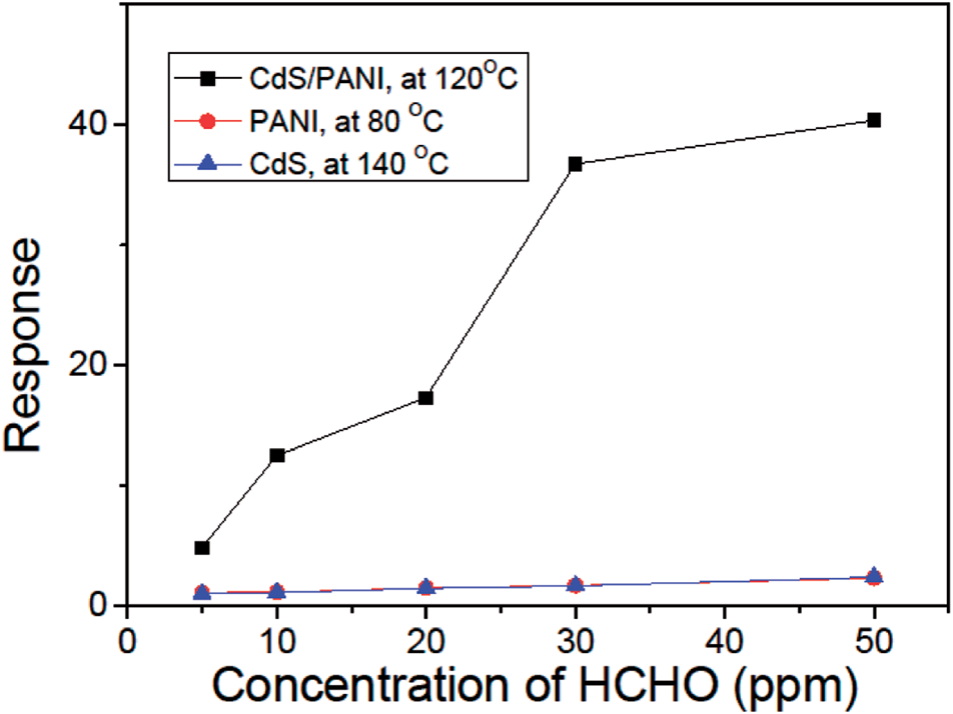
Relationship between HCHO gas concentration and the response of sensors of PANI, CdS and CdS/PANI nanorods sample-based at their respective optimum working temperature.

**Fig. 10:**
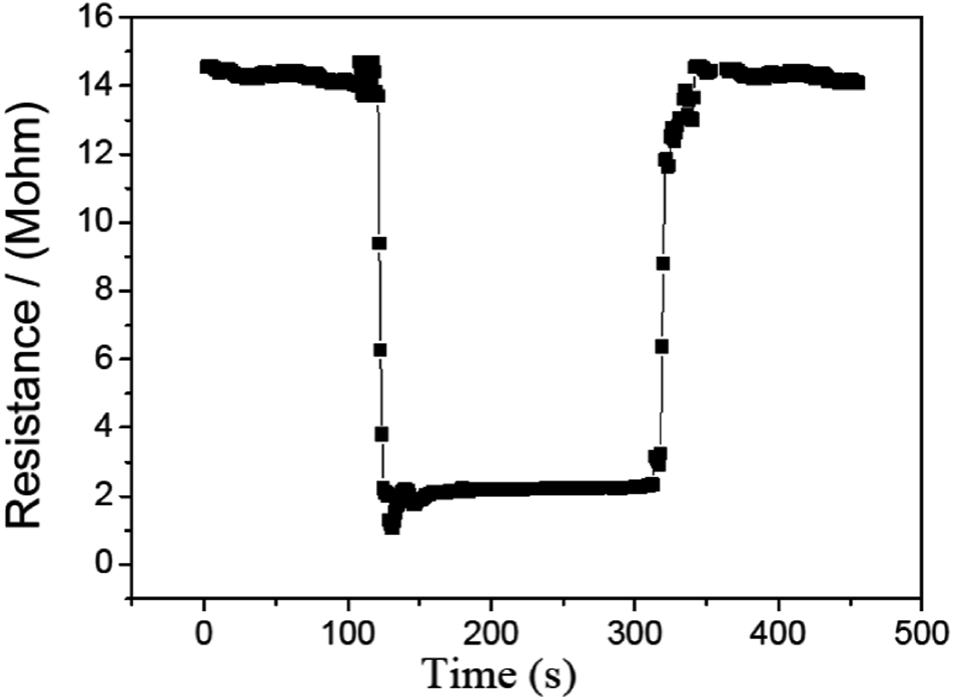
The survey of the response and recovery behavior for the CdS/PANI nanorods-based sensor to 10 ppm at 120 °C.

Fig. 10 shows the survey of the response and recovery behavior for the CdS/PANI nanorods sample to 10 ppm HCHO gas at 120 °C. It can be seen that the resistance curve decreases dramatically down the injection of the tested gas and rises to the initial value rapidly with the remove of the tested gas. These results confirm that our sample had a typical behavior of n-type semiconductor. As shown in Fig. 9, the sensor shows good response–recovery characteristic when detecting 10 ppm HCHO gas. The response and recovery time was about 25 S and 30 S, respectively.

**Fig. 11:**
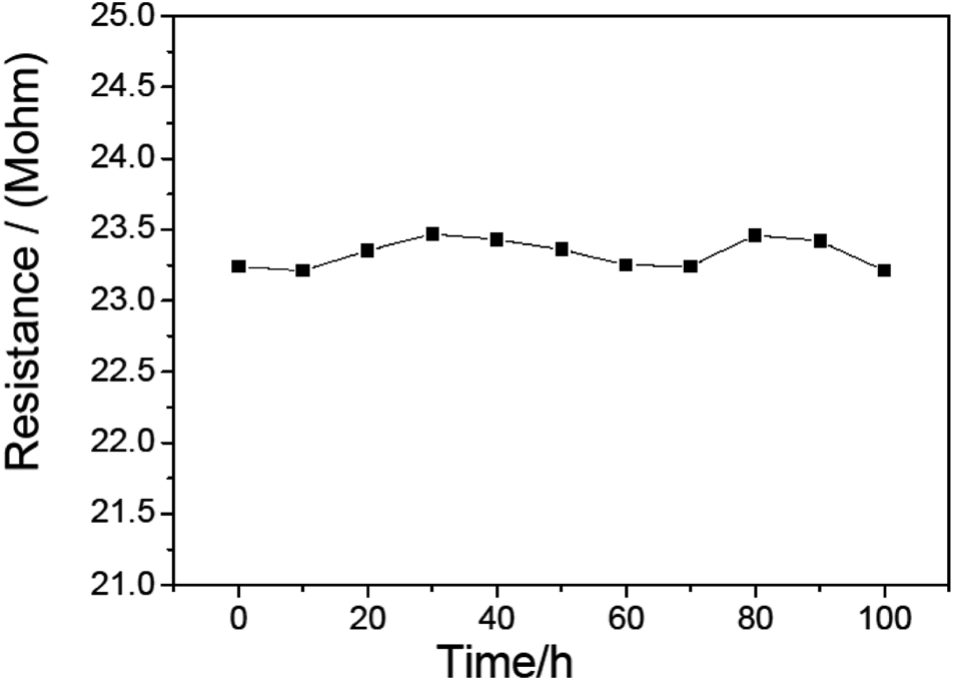
Resistance stability of CdS/PANI nanorods-based sensor.

To study the stability of this CdS/PANI nanorods material based sensor. We continuously measured the resistance of this sensor for 100 h at 120 °C. As is shown in Fig. 11, we found that its resistance is stable except a small variation of about 0.26 M within 100 hours, and after several months its sensitivity to 500 ppm HCHO gas still reached 100.2. This sensor showed good stability and durability.

## 4. Conclusions

A novel composite, CdS nanorods growing on a polyaniline particles surface (CdS/PANI) with a hexagonal wurtzite structure phase, was prepared using a hydrothermal synthesis method. Compared to pure CdS nanorods, SEM shows that CdS/PANI consists of sea urchin-like nanorods of about 200–500 nm in length and about 50 nm in diameter. Moreover, the aromatic ring bending vibration peaks, C–N stretching and C–H flexural vibration absorption band of the benzene ring of CdS/PANI from the FTIR spectra are much weaker than those of PANI and the corresponding peaks are shifted to a higher wavenumber. In addition, the stretching frequency of the Cd–S bond for CdS/PANI moved from 630 cm^−1^ to 674 cm^−1^. The CdS/PANI-based sensor showed an excellent response to low concentration formaldehyde gas in a wide temperature range of 80–140 °C, and moreover the sensor showed good stability and durability of the performance at operating temperature. The highest response of the CdS/PANI could reach about 4.8 to 5 ppm formaldehyde gas at 120 °C. The response and recovery times of the sensor based on CdS/PANI were about 25 s and 30 s to 10 ppm formaldehyde gas, respectively.

## Conflicts of interest

There are no conflicts to declare.

## Supplementary Material
